# Effect of home-based Tai Chi, Yoga or conventional balance exercise on functional balance and mobility among persons with idiopathic Parkinson’s disease: An experimental study

**DOI:** 10.1142/S1013702520500055

**Published:** 2020-02-20

**Authors:** Arva Khuzema, A. Brammatha, V. Arul Selvan

**Affiliations:** 1 KMCH College of Physiotherapy, Avinashi Road, Coimbatore, Tamil Nadu, India; 2 Kovai Medical Centre and Hospital, Avinashi Road, Coimbatore, Tamil Nadu, India; arva24x7@gmail.com; brammatha@yahoo.co.in

**Keywords:** Parkinson’s disease, Tai Chi, yoga, balance, home-based setting

## Abstract

**Background:**

Individuals with Parkinson’s disease (PD) invariably experience functional decline in a number of motor and non-motor domains affecting posture, balance and gait. Numerous clinical studies have examined effects of various types of exercise on motor and non-motor problems. But still much gap remains in our understanding of various therapies and their effect on delaying or slowing the dopamine neuron degeneration. Recently, Tai Chi and Yoga both have gained popularity as complementary therapies, since both have components for mind and body control.

**Objective:**

The aim of this study was to determine whether eight weeks of home-based Tai Chi or Yoga was more effective than regular balance exercises on functional balance and mobility.

**Methods:**

Twenty-seven individuals with Idiopathic PD (Modified Hoehn and Yahr stages 2.5–3) were randomly assigned to either Tai Chi, Yoga or Conventional exercise group. All the participants were evaluated for Functional Balance and Mobility using Berg Balance Scale, Timed 10 m Walk test and Timed Up and Go test before and after eight weeks of training.

**Results:**

The results were analyzed using two-way mixed ANOVA which showed that there was a significant main effect for time as F (1, 24) =74.18, p=0.000, $\eta p^{2}=0.76$ for overall balance in Berg Balance Scale. There was also significant main effect of time on mobility overall as F(1, 24) =77.78, p=0.000, $\eta p^{2}=0.76$ in Timed up and Go test and F(1, 24) =48.24, p=0.000, $\eta p^{2}=0.67$ for 10 m Walk test. There was a significant interaction effect for time×group with F(2, 24) =8.67, p=0.001, $\eta p^{2}=0.420$ for balance. With respect to mobility, the values F(2, 24) =5.92, p=0.008, $\eta p^{2}=0.330$ in Timed Up and Go test and F(2, 24) =10.40, p=0.001, $\eta p^{2}=0.464$ in 10 m Walk test showed a significant interaction. But there was no significant main effect between the groups for both balance and mobility.

**Conclusion:**

The findings of this study suggest that Tai Chi as well as Yoga are well adhered and are attractive options for a home-based setting. As any form of physical activity is considered beneficial for individuals with PD either Tai Chi, Yoga or conventional balance exercises could be used as therapeutic intervention to optimize balance and mobility. Further studies are necessary to understand the mind–body benefits of Tai Chi and Yoga either as multicomponent physical activities or as individual therapies in various stages of PD.

## Introduction

Parkinson’s disease (PD) is a chronic progressive neurodegenerative disorder of insidious onset characterized by the presence of predominantly motor symptoms and a diversity of non-motor symptoms.^1^ The Clinical Diagnostic Criteria proposed by UK Parkinson’s Disease Society Brain Bank is Bradykinesia (slowness of initiation of voluntary movement with progressive reduction in speed and amplitude of repetitive actions); And at least one of the following: Muscular rigidity, 4–6 Hz resting tremor, Postural instability not caused by primary visual, vestibular, cerebellar, or proprioceptive dysfunction.^2^

PD is a universal disorder, with a crude incidence rate of 4.5–19 per 100,000 population per year and the total Daily Adjusted Life Years (DALY) globally in 2015 was 1.76 million (0.12%) and is expected to increase up to 2.01 million (0.13%) in 2030.^3^ In a door-to-door survey of the Parsi community in Bombay, the crude prevalence estimates were 328 per 100,000.^4^

The basal ganglia, a key pathologic structure in PD, is involved in control of balance via the thalamic-cortical-spinal loops, the brainstem pedunculopontine nucleus and the reticulospinal system. The basal ganglia is involved in controlling the flexibility of postural tone, scaling up the magnitude of postural movements, selecting postural strategies for environmental context (central set), and automatizing postural responses and gait. Early in the disease, dopamine-sensitive bradykinesia and rigidity progressively affect balance and gait and later in the disease, dopamine insensitive-balance problems like impaired kinaesthesia, inflexible postural set, lack of automaticity, and executive dysfunction aggravate the balance and gait impairments.^5^

Cochrane’s updated review in 2014 included 43 trials which highlight that a wide range of physiotherapy techniques have been tested to treat PD. Considering the small number of participants, the wide variety of physiotherapy interventions and the outcomes assessed, there is insufficient evidence to support the use of one approach of physiotherapy intervention over another for the treatment of PD.^6^ There is a moderate evidence that physical activity and exercise will result in improvements in postural instability outcomes and to improve balance task performance in persons with mild to moderate PD.^7^ A recent survey showed that Tai Chi and Yoga were the second most prevalent complimentary therapies utilized by individuals with PD and the perceived effectiveness of Yoga and Tai Chi were reported to be 73.8% and 60.9%, respectively.^8^

Tai Chi is a traditional Chinese martial art that involves slow and graceful movements that can improve postural balance, flexibility, and mood.^9^ Tai Chi, as a mind–body exercise, consists of a series of dance-like movements linked in a continuous sequence, flowing slowly and smoothly from one movement to another that emphasizes weight transfer and movement of the body. The Tai Chi stresses weight shifting that trains the ankle strategy to effectively move the person’s center of gravity toward the limits of stability, also by alternating between a narrow stance and a wide stance, the dorsiflexor and plantar flexors are strengthened.^10^

Previous studies had reported that mind–body exercises like Tai Chi showed improvement in motor and non-motor symptoms.^11^ Tai Chi has reported to have promising improvement in mobility and balance, also it is considered safe and popular among individuals with PD at an early stage along with medications.^12^ A meta-analysis concluded that improvement in balance was greater for Tai Chi plus medication than other exercise plus medication and medication alone.^13^ But studies also have found that there was no significant difference in the gait velocity between Tai Chi and other exercises.^10,13^

Yoga is a popular mind-and-body practice which originated in ancient India. It concentrates on meditation, breathing, and postures. The control of posture practice in Yoga involves stretch and balance while maintaining a stable sitting or standing position. The reported benefits of Yoga training for healthy populations include improving muscle strength and endurance, muscle power, flexibility, balance and coordination, and health-related functions.^14^ Yoga may also have psychosocial benefits through prevention and control of common health and emotional problems linked with aging.^15^ A 12-week study on 13 participants with PD reported that there was a significant improvement in balance, strength, flexibility, and range of motion.^16^ A pilot study reported that 3-month Yoga program significantly reduced bradykinesia and rigidity, and increased muscle strength and power in individuals with PD.^17^

Based on an analysis of dose for intervention prescription, it was found that for home-based exercise for people with Idiopathic Parkinson’s disease, a minimum 150 min per week for at least six weeks improved balance-related activities.^18^ Although India’s ancient religious text brought forth the teachings and practice of Yoga, over time, it has been described as a way of uniting body and mind which is yielding therapeutic benefits. Based on the available literature, both therapies can be considered as an adjuvant for the patients with various neurological disorders. However, there is no recent literature evaluating the effects of home-based Tai Chi or Yoga on individuals with PD. So, this study focused on comparing the effects of home-based Tai Chi, Yoga or Conventional balance exercise program on balance and functional mobility among persons with idiopathic PD.

## Methods

### Participants

G-power software (version 3.1.9.2) was used to calculate the number of patients required for this study to achieve a significant level of 0.05, power of 0.95, and effect size of 0.73. To achieve the required power, 27 patients were included in this study. Inclusion criteria was (a) Subjects within age 60–85 years including both male and female. (b) Subjects who were in stage 2.5 (Mild bilateral disease with recovery on pull test) and 3 (Mild to moderate bilateral disease; some postural instability; physically independent) of Modified Hoehn and Yahr’s Parkinson’s stage. (c) Patients who were able to understand and follow the instructions. (d) Patients who were interested to participate and were not undergoing any other treatment other than medication.

Participants were excluded if they had (a) Severe co-morbidity influencing mobility or life-threatening disease. (b) Not interested to participate in any form of exercises. (c) Visual and vestibular disorder affecting balance. (d) History of osteoporosis, fracture or ankle instability, falls. (e) No care giver supervision or support.

**Fig. 1.**
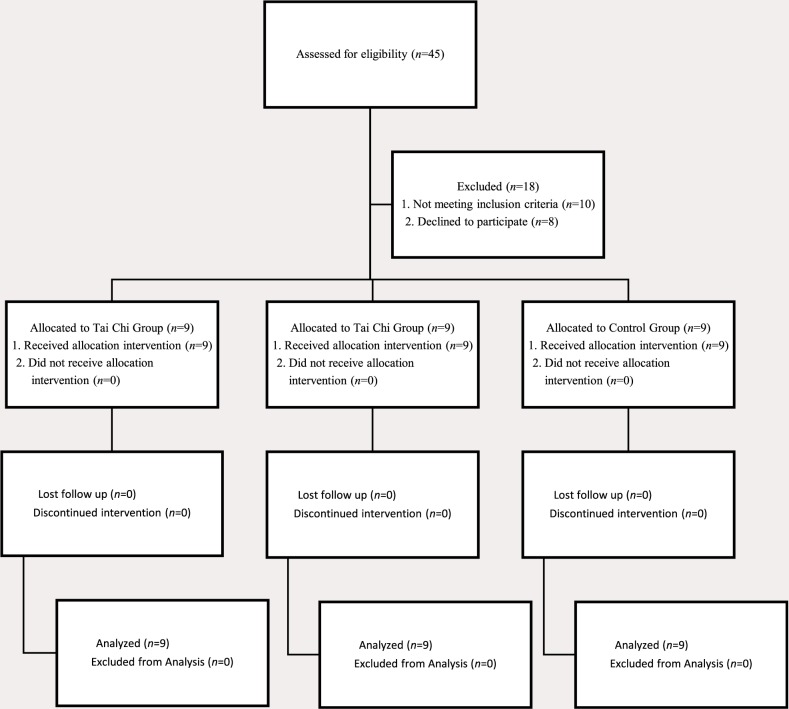
Study flowchart.

All patients underwent a routine neurological assessment and were also assessed with Unified Parkinson’s Disease Rating Scale (UPDRS) and sternal nudge test to classify the Modified Hoehn and Yahr’s stage of PD in the “on” phase of medication by the Neurologist with a 25 years of experience and Movement disorder specialist at the Department of neurology, Kovai Medical Center and Hospital and then were referred to the Physiotherapist for the study. Twenty seven subjects who satisfied the selection criteria participated in this study and a written informed consent was obtained. These 27 patients were blinded to the groups and were randomly allocated by the alternate number method to the three groups. 9 patients were allocated to Group A: (Experimental Group I) who received Home-based Tai Chi exercise program, 9 patients were allocated to Group B: (Experimental Group II) who received Home-based Yoga exercise program and 9 Patients were allocated to Group C: (Control Group) who received Home-based General Balance exercises (Fig. 1).

### Intervention

Tai Chi, Yoga and Conventional exercise programs were designed according to expert opinion and from available literature for a duration of eight weeks. Participants in Tai Chi group were taught exercises by the Physiotherapist under the guidance of the Tai Chi instructor with an experience of 10 years. Yoga was taught to the participants belonging to the Yoga group by the Physiotherapist who was certified in Yoga. General balance exercises were taught to the participants in the control group by the Physiotherapist. The first session was conducted at the Department of Physical Therapy, Kovai Medical Center and Hospital, India. The subjects were given an exercise pamphlet (supplement) and a record sheet was also provided to rate their adherence towards the exercise intervention and to mention about any difficulties they faced during the exercises which was collected after eight weeks. All the instructions and explanations to the participants were given in the presence of a family member who aided the home intervention. They were advised to do the exercises for a five days/week at a slow and comfortable pace and within the intensity ranges of 11–15 (light to somewhat hard) on the Borg Rating of Perceived Exertion Scale.^19^ The subjects were advised to perform all the exercises on non-slippery floor and during the “on” phase of the medication that is within one to two hours after taking their medications preferably in the morning itself. The communication with the patient and the family member was established by means of a telephone call made every third day till the end of intervention. The patient and the family member were advised to contact at any time during the intervention in case of any difficulty.

### Tai Chi exercise program

**Fig. 2.**
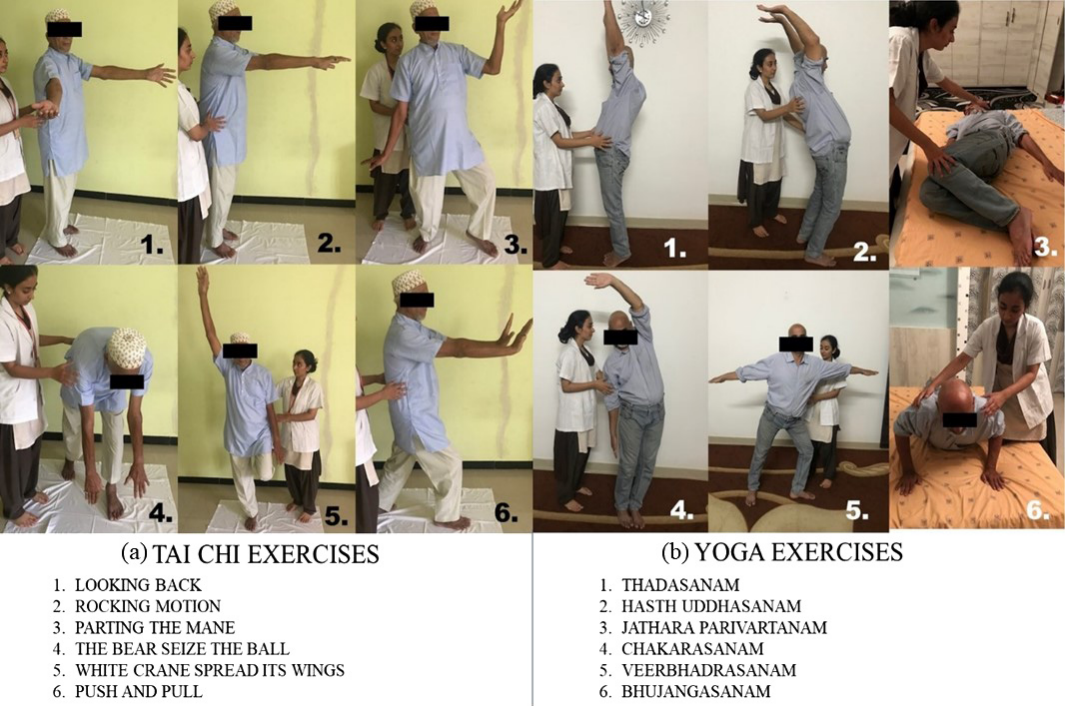
Figure illustrating the exercises given to (a) Tai Chi Group and (b) Yoga Group.

Each session of Tai Chi exercise program lasted for about 30–40 min and it included six exercise poses. Each pose (Fig. 2(a)) was repeated about five times initially and were gradually increased to 10 repetitions according to the subject comfort. All the exercises were performed in a slow pace with abdominal breathing pattern.

### Yoga exercise program

Each session of the Yoga exercise program lasted 30–40 min and included six poses. Subjects were advised to breathe deeply and effortlessly with inhalation through nose and exhalation through mouth during the exercise. Each pose (Fig. 2(b)) was repeated five times initially and gradually increased to 10 repetitions according to the subject’s preference.

### Conventional balance exercise program

It included six conventional balance exercises which were standing back extensions, standing trunk rotations, backward walking, side-ways walking, tandem walking and single limb stance. Each exercise was repeated five times initially and gradually increased to 10 repetitions according to the subject’s comfort. Subjects were advised to perform 10 steps in each repetition of Backward walking, Side-ways walking and Tandem walking. The exercise session lasted for about 40–45 min.

### Outcome measures

All the outcome measures were assessed by the researcher who was not blinded. The balance was assessed by the Berg Balance Scale which comprises of 14 items on a five-point scale, ranging from 0–4.^20^ “0” indicates the lowest level of function and “4” the highest level of function for a total score of 56. The functional mobility was assessed by the Timed 10-m Walk test and Timed Up and Go test. In Timed 10-m Walk test, the subjects were instructed to walk on a 10-meter long path and the time was measured in seconds for the intermediate 6 m (19.7 feet) to allow for acceleration and deceleration.^21^ The Timed Up and Go test begins with the subject comfortably sitting on a standard chair with arm rest and then the subjects were instructed to stand up from the chair, walk to the line on the floor three meters away, turn around and walk back to the chair and sit down at a self-selected pace, the time duration in seconds for this was noted.^22^ The outcome measures were obtained at baseline and after eight weeks as patients came for review to the hospital in the same setting.

## Data Analyses

Data analysis was performed using SPSS Software (version 25). Mixed model (3×2) ANOVA was conducted that examined the effect of eight weeks Tai Chi, Yoga or Conventional Balance exercises on Balance and Mobility. Alpha level of significance was set at 0.05.

## Results

Twenty-seven individuals with Idiopathic PD were assigned to either Tai Chi, Yoga or conventional exercise group who participated in this study. There was no difference in the baseline for age, UPDRS motor score, Modified Hoehn and Yahr’s stages, sex, time with PD, Balance score, timed up and go time and 10 m walk time (Table 1) analyzed using Mixed Model ANOVA. The Two-way mixed ANOVA analysis (Table 2) for overall balance in Berg Balance Scale scores showed that there was a significant main effect for time as F(1,24)=74.18, p=0.000, $\eta p^{2}=0.76$. There was also a significant main effect of time on mobility overall as F(1, 24) =77.78, p=0.000, $\eta p^{2}=0.76$ in Timed Up and Go test and F(1, 24) =48.24, p=0.000, $\eta p^{2}=0.67$ for 10 meter walk test.

**Table 1.** Participant (n=27) characteristics at baseline of the eight weeks Tai Chi, Yoga or General balance exercise study.

		Sex		Time with	Motor subscale	Modified H&Y stage				10 mWt
Groups	Age (in years)	M	F	PD (years)	score (UPDRS)	2.5	3	BBS score	TUG time (s)	time (s)
Tai Chi	72 ± 5.22	6	3	5.67 ± 2.33	17.22 ± 6.53	3	6	40.889 ± 6.94	16.328 ± 5.41	8.981 ± 2.33
Yoga	68.11 ± 4.23	6	3	6.2 ± 1.67	17.67 ± 6.30	3	6	44.222 ± 4.79	20.094 ± 13.18	10.497 ± 8.27
Control	70.89 ± 6.01	7	2	5.23 ± 3.12	20.22 ± 6.72	4	5	41.000 ± 9.19	16.203 ± 7.18	7.872 ± 2.98

*Notes*: PD- Parkinson’s Disease; H&Y- Hoehn & Yahr Stage; BBS- Berg Balance Scale: TUG- Timed Up and Go; 10 mWt- 10 m Walk test.

**Table 2.** Pre-intervention values, Post-intervention values and p values assessed by Mixed Model ANOVA for Berg Balance Scale, Timed Up and Go and 10-m walk test.

Outcome measure	Treatment groups	Pre-test means with standard deviation	Post-test means with standard deviation	p=Main effects for time	p=Main effects for groups	p=Time and group interaction
Berg Balance Scale (score)	Tai Chi	40.889 ± 6.94	53.333 ± 1.32	0.000*	0.566	0.001*
	Yoga	44.222 ± 4.79	48.000 ± 4.69			
	Control	41.000 ± 9.19	47.333 ± 7.70			
Timed Up and Go (Seconds)	Tai Chi	16.328 ± 5.41	13.000 ± 4.4	0.000*	0.507	0.008*
	Yoga	20.094 ± 13.18	18.700 ± 13.54			
	Control	16.203 ± 7.18	14.822 ± 6.5			
10-m Walk test (Seconds)	Tai Chi	8.981 ± 2.33	7.023 ± 2.13	0.000*	0.053	0.001*
	Yoga	10.497 ± 8.27	9.894 ± 8.2			
	Control	7.872 ± 2.98	7.195 ± 2.79			

*Note*: *Level of significance p≤0.05.

There was a significant interaction effect for time×group with F(2, 24) =8.67, p=0.001, $\eta p^{2}=0.420$ for balance. With respect to mobility, the values F(2, 24) =5.92, p=0.008, $\eta p^{2}=0.330$ in Timed Up and Go test and F(2, 24) =10.40, p=0.001, $\eta p^{2}=0.464$ in 10 m Walk test showed a significant interaction.

There was no significant main effect for group with F(2, 24) =0.583, p=0.566, $\eta p^{2}=0.046$ on balance. Similarly, there was no significant main effect found between groups for mobility with F(2, 24) =0.699, p=0.507, $\eta p^{2}=0.055$ in Timed Up and Go test and F(2, 24) =0.667, p=0.523, $\eta p^{2}=0.053$ in 10 m Walk test. Further, *Post Hoc* analysis was not done as there was no significant group effect.

**Fig. 3.**
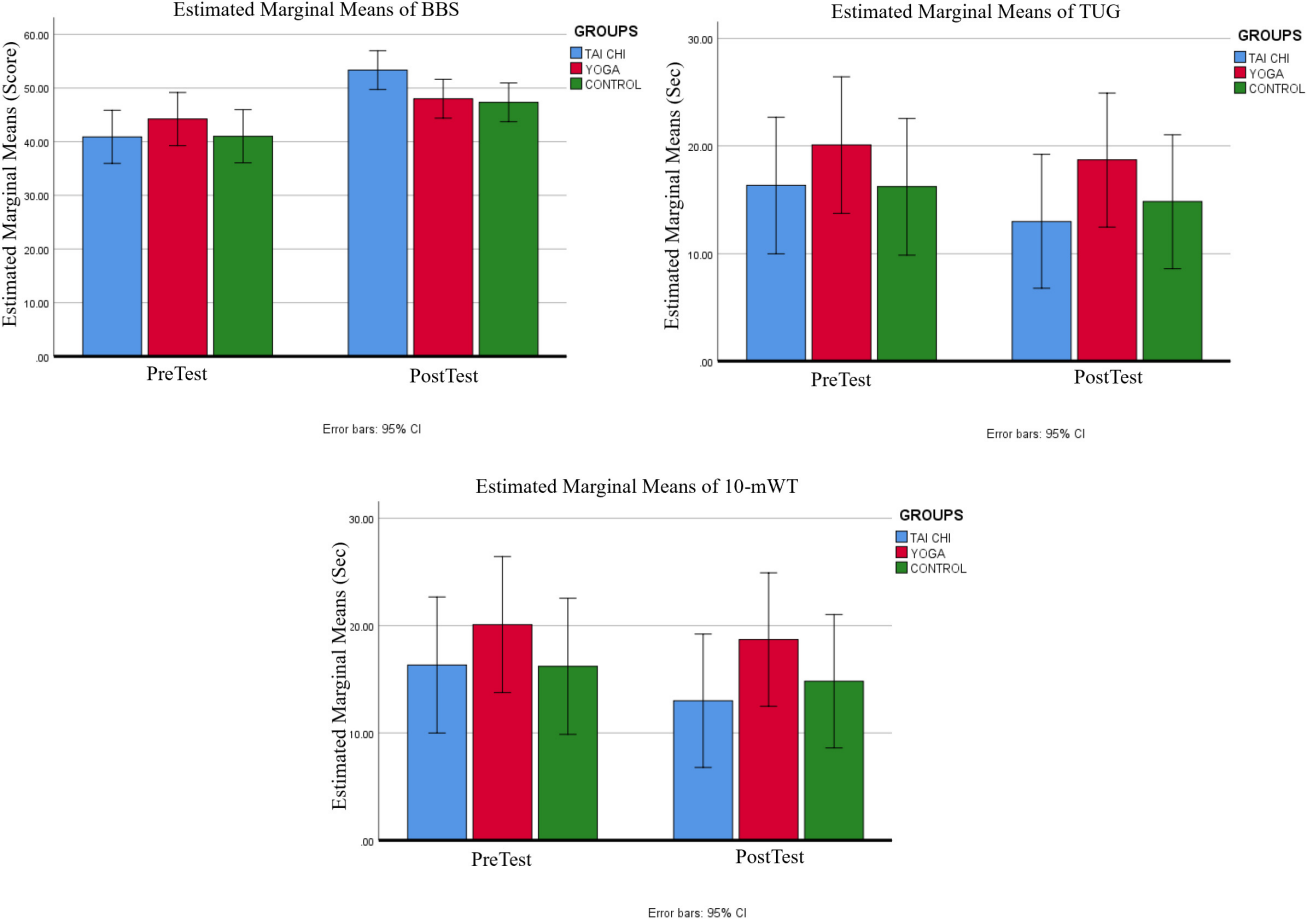
Clustered bar graph depicting mean and standard deviation before and after eight weeks among three groups for Berg Balance Scale, Timed Up and Go and 10-m Walk test among participants.

The mean Berg Balance score increased significantly by 26.414%, 8.193% and 14.339% in Tai Chi, Yoga or Conventional balance exercise group, respectively (p<0.05). The mean Timed up and go time decreased significantly by 22.695%, 7.187% and 8.902% in Tai Chi, Yoga and Conventional balance exercise group, respectively (p<0.05). The mean 10-m Walk Time decreased significantly by 24.469%, 5.914% and 8.986% in Tai Chi, Yoga and Conventional balance exercise group, respectively (p<0.05) (Fig. 3).

All the subjects reported good adherence with a 92.78% compliance in Tai Chi group, 90.28% compliance in Yoga group and 71.94% compliance in control group. All the participant (100%) completed the program with 0% adverse events, injuries or falls.

## Discussion

The home-based Tai Chi, Yoga or Conventional balance exercise program for eight weeks (40 sessions) showed no significant difference in balance and functional mobility between the three groups. However, all the three groups showed statistically significant improvement after eight weeks in balance and functional mobility.

Xiaojia Ni *et al.*^13^ in their meta-analysis reported mean difference (MD=4.25, 95% CI 2.85–5.86) in the BBS score. In this study, the mean difference in the BBS score between Tai Chi and Yoga group was (MD=5.33, 95% CI 0.86–11.52) and between Tai Chi and Control group was (MD=6.00, 95% CI 0.19−12.19). Li *et al.*^10^ found that Tai Chi training reduces balance impairment in patients with mild to moderate Parkinson’s Disease. Choi *et al.*^9^ found significant interaction effects in balance with 36 sessions of therapeutic Tai Chi given for 12 weeks. Later, Gao *et al.*^23^ found that 26-week Tai Chi training improved more than control group on Berg Balance Scale, but not on Timed Up and Go Test. However, our results showed that there was statistically significant difference after eight weeks on both balance and mobility in all the three groups. However, there was no statistically significant difference in balance and mobility among the three groups.

In 2017, review by Song *et al.*^11^ and in 2014, review by Yang *et al.*^24^ found improvement in balance (Effect size=0.544, p<0.0001) and efficacy of balance (SMD-1.22, 95% CI; 0.80–1.65, P<0.00001), respectively. Berg Balance Scale (BBS) is mostly used to assess balance function in Parkinson’s disease. It has been previously reported that the Minimal Clinical Relevant Difference of the BBS is the improvement by five points.^25^ In this study, a clinical improvement of 12.44 ± 6.19 points in the Tai Chi group was observed for balance after 40 sessions in eight weeks which is more than needed Minimal Clinical Relevant Difference.

The Tai Chi protocol stresses weight shifting and ankle sway to effectively move the person’s center of gravity toward the limits of stability, alternating between a narrow stance and a wide stance to continually change the base of support, increasing support-leg standing time, engaging rotational trunk movements with upright posture, and performing heel-to-toe (forward) and toe-to-heel (backward) stepping movements to strengthen dorsiflexion and plantar flexion. Tai Chi also proposes improved flexibility and muscle strength.^10^ Although these improvements indicate that Tai Chi would be effective in enhancing neuromuscular rehabilitation, the mechanisms behind the therapeutic change in participant’s motor control and mobility remain less understood and needs future exploration.

The Tai Chi group showed an increase in mean BBS score by 26.414% while the Yoga group showed an increase in BBS score by only 8.193%. This could be explained in many ways. The first is Tai Chi that has many styles. In this study, Tai Chi was based on Qigong style, which was considered easy to follow and had different moves challenging balance.^26^ Similarly, the Yoga group in this study had Thadasanam, Hastha Uddhanasanam, Chakrasanam, Veerabhadrasanam, Jathara Parivarthanam and Bhujangasanam Postures, which was mostly static and might not be sufficient in providing dynamic postural challenges. Second, different forms of Yoga and Tai Chi exist, varying in intensity as well as benefits. There is no stage specific standard Yoga protocol which could be tested against other complementary or mind–body therapies.^27^ Third, Yoga could be considered as a spiritual intervention by the participants and Tai Chi as wellness intervention program. This study focused only on balance and mobility, not on non-motor symptoms like mood or depression which could have been influenced by Yoga. Cheung *et al.*^28^ reported that Yoga is a safe, feasible and acceptable complimentary method for improving motor function in individuals with mild to moderate Parkinson’s disease. However, longer duration or different set of Yoga patterns may be necessary for improvement in motor and non-motor functions in individuals with PD.

The protocol of the conventional balance exercise focused on the exercise that require the control of the body’s center of mass while performing destabilizing movements and during reduction in base of support. Both static and the dynamic balance exercises were included in the conventional balance exercise program like single leg standing, standing back extension, standing trunk rotation, backward walking, heel walking and tandem walking. However, the conventional exercise group showed an increase in mean BBS score by 14.339% when compared to the Tai Chi group which showed an increase in mean BBS score by 26.414%. This could be because the exercises may not be sufficiently challenging for the subjects.

Regarding mobility component, Song *et al.*^11^ in their 2017 review concluded that Tai Chi groups showed significant improvement in TUG Score when compared to the control groups, with a small effect size (Hedges’s g=−0.341, 95% CI −0.578 to –0.104, p=0.005). However, Choi *et al.*^9^ concluded there was no significant change in TUG scores in both Tai Chi and Control group after 12 weeks of training. On the other hand, the results of this study showed that there was statistically a significant difference in TUG scores in all the three groups. But there was no statistical significant differences in TUG scores after eight weeks among the three groups. Our finding in this study by the 10 m Walk test showed that there was a statistically significant difference in the scores after eight weeks of training in all the three groups, but no statistically significant difference was found among the three groups. The exercise protocol consisted of challenging movements in multiple direction which require more complex coordination that might have contributed to improved mobility.

Considering the difficulties in transport and cost, further follow up of participants were not carried out in this study. The participants needed moderate supervision during the initial period but however were able to perform all the exercise independently once they became confident.

Both Tai Chi and Yoga are increasingly gaining popularity as preferred Physical Activity. The “2018 Physical Activity Guidelines for Americans” recommends Tai Chi and Yoga as muscle strengthening exercise. This study found that though with eight weeks of training Tai Chi, Yoga or Conventional balance exercises did not show statistical significance among the three groups, there were beneficial effects on balance and mobility. Either Tai Chi or Yoga could be a good exercise strategy which individuals with PD can choose according to their preference and interest.

## Conclusion

Both home-based Tai Chi or Yoga could be a potential therapy for improving Balance and Functional mobility for individuals with mild to moderate Idiopathic Parkinson’s disease. These exercise programs are well adhered by the patient and can be an attractive option. Further, the effect of these therapies on various Hoehn and Yahr’s stages of disease, duration and progression must be studied. Also, long term follow-up and large-scale studies are required to gain better insight and understanding.

### Limitations

There were several limitations of this study that includes being mind and body exercises, the psychological aspect of Tai Chi and Yoga were not assessed. The researcher was not blinded to the groups of participants. The exercises were needed to be performed under supervision for an initial period. The entire study was performed in the “on” phase. A long-term follow-up was not done. Exercises for each group was established from previous studies, there was no stage specific exercise sets available.

### Suggestions

Future research should focus on the effects of Tai Chi and Yoga therapy on non-motor symptoms of Parkinson’s disease. Integrated exercise program for Balance can be established which can include either Tai Chi and Yoga together or separately. Future research should also focus on the effects of supervised versus unsupervised mode of exercise or group versus individualized therapy.

## Acknowledgments

We would like to thank Dr. Edmund M. D’couto and Dr. Vennila for their assistance. We would like to acknowledge Mr. Kathiresan for supervising Tai Chi exercise program.

## Conflict of Interest

The authors have no conflict of interest.

## Funding/Support

This research did not receive any specific grants from any commercial, public, or non-profit funding agencies.

## Author Contributions

AK and AB were involved in study conception and design. AK and AS performed data acquisition and test procedures. AK and AB performed the data analysis and/or interpretation. AK and AB wrote the first draft of this paper and all the authors revised it critically for important intellectual concept. All authors have given final approval of the version to be published.
